# Functional L-Arginine Derivative as an Efficient Vector for Intracellular Protein Delivery for Potential Cancer Therapy

**DOI:** 10.3390/jfb14060301

**Published:** 2023-05-30

**Authors:** Xiao He, Yannv Qu, Su Xiong, Zhiru Jiang, Yaqin Tang, Fei Yan, Yuanfei Deng, Yansun Sun

**Affiliations:** 1 Department of Geriatrics, Peking University Shenzhen Hospital, Shenzhen 518036, China; 2 Center for Cell and Gene Circuit Design, CAS Key Laboratory of Quantitative Engineering Biology, Shenzhen Institute of Synthetic Biology, Shenzhen Institutes of Advanced Technology, Chinese Academy of Sciences, Shenzhen 518055, China; fei.yan@siat.ac.cn; 3 Chongqing Key Laboratory of Medicinal Chemistry and Molecular Pharmacology, Chongqing University of Technology, Chongqing 400054, China; susu@stu.cqut.edu.cn (S.X.); tangyaqin@cqut.edu.cn (Y.T.)

**Keywords:** self-assembly, L-arginine derivatives, DBCO, click chemical reaction, protein delivery

## Abstract

The utilization of cytosolic protein delivery is a promising approach for treating various diseases by replacing dysfunctional proteins. Despite the development of various nanoparticle-based intracellular protein delivery methods, the complicated chemical synthesis of the vector, loading efficiency and endosomal escape efficiency of proteins remain a great challenge. Recently, 9-fluorenylmethyloxycarbonyl (Fmoc)-modified amino acid derivatives have been used to self-assemble into supramolecular nanomaterials for drug delivery. However, the instability of the Fmoc group in aqueous medium restricts its application. To address this issue, the Fmoc ligand neighboring arginine was substituted for dibenzocyclooctyne (DBCO) with a similar structure to Fmoc to obtain stable DBCO-functionalized L-arginine derivative (DR). Azide-modified triethylamine (crosslinker C) was combined with DR to construct self-assembled DRC via a click chemical reaction for delivering various proteins, such as BSA and saporin (SA), into the cytosol of cells. The hyaluronic-acid-coated DRC/SA was able to not only shield the cationic toxicity, but also enhance the intracellular delivery efficiency of proteins by targeting CD44 overexpression on the cell membrane. The DRC/SA/HA exhibited higher growth inhibition efficiency and lower IC_50_ compared to DRC/SA toward various cancer cell lines. In conclusion, DBCO-functionalized L-arginine derivative represents an excellent potential vector for protein-based cancer therapy.

## 1. Introduction

Protein drugs have been the primary treatment strategy for autoimmune diseases and cancer induced by dysfunctional proteins [1]. Compared to the traditional small-molecule chemical drugs, protein drugs have numerous outstanding advantages, including safety, high efficiency and specificity. In recent years, the best-selling and most popular drugs have almost all been proteins, such as monoclonal antibodies, cytokines and functional hormones [2]. However, these protein drugs play an important role regarding extracellular receptors, and intracellular protein delivery has potential value for proteins acting on cytosolic targets [3]. Due to uncertain charge, poor membrane permeation and the instability of protein, it remains challenging to deliver proteins into cells without losing biological functions. Therefore, it is important for protein-based disease therapies to develop novel protein delivery strategies [4,5,6,7].

To solve the challenge of how to achieve intracellular protein delivery, researchers have developed and designed various methods, including: (i) membrane perturbation-based physical strategies, such as electroporation, microinjection and microfluidics [8,9]; (ii) the attachment of the cargo protein to the cell-permeated ligands, such as cell-penetrating peptides (TAT, cTAT, Pep-1) and cell-penetrating poly(disulfides) (CPD) [10,11,12,13,14,15,16,17,18]; and (iii) nanotechnology-based delivery vectors, such as nanogels, metal–organic frameworks, exosomes, liposomes and polymers [19,20,21,22]. Impressively, the organic nanovectors can be adjusted and designed upon the properties of proteins containing diverse amino acids of different size and charges. For the efficient internalization of native proteins into cells, proteins should be integrated into nanovectors, which have high loading efficiency and anti-degradation ability. Subsequently, the delivered protein was released in the cytosol with vector-assisted efficient endo/lysosomal escape [23]. Despite significant progress in protein delivery, recent strategies have several disadvantages, such as protein chemical modification, the complicated synthesis process of vectors and the application of organic solvent damaging protein [24,25,26,27,28]. Therefore, it is necessary to design novel nanovectors with high loading efficiency, simple preparation processes and no requirement for protein modification.

Self-assembly plays an important role in retaining the function of biological systems through promoting the formation of delicate and precious structures. For example, self-assembly can result in the tertiary and quaternary structures of proteins with driven force via hydrogen bonds and hydrophobic and electrostatic interaction [29]. Inspired by natural self-assembly behavior in protein structures, macromolecules can be employed as building blocks to construct artificial functional materials applied to the different fields of drug delivery, energy and biology via biomimetic design [30,31,32]. For instance, the representative macromolecules, such as proteins or peptides, are the basic self-assembled building blocks for the formation of ordered biological structures, such as α-helixes, β-sheets and microtubules [33,34,35,36]. Proteins and peptides with self-assembly abilities can supply the biological system with promoted mechanical force and enhanced stability. In addition, single amino acids and their derivatives (such as Fmoc-modified amino acid) possess advantages such as simple preparation processes, accurate constructions and easy repetition [37,38]. They are applied to self-assemble into the formation of functional supramolecular nanocarriers for efficient drug delivery, which offer a novel strategy for the improvement of drug administration in clinical therapy. Therefore, it has promising potential to develop and design self-assembled amino acid derivatives applied for the efficient encapsulation and intracellular delivery of proteins.

**Figure 1:**
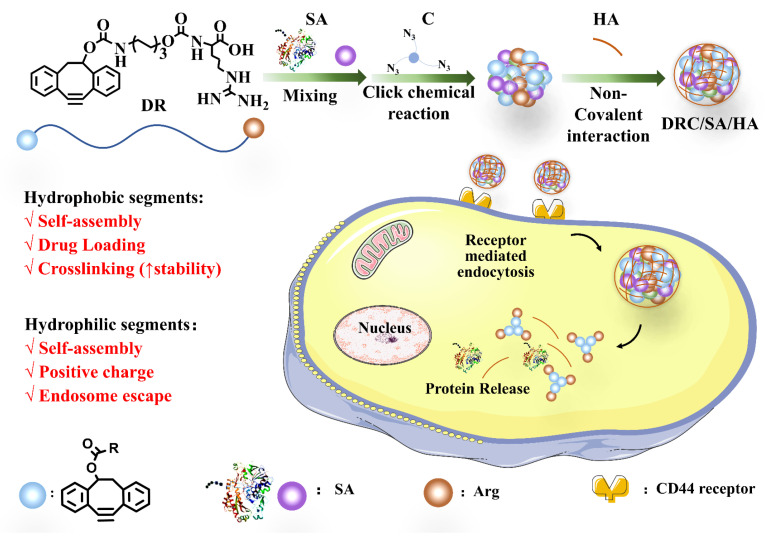
Schematic illustration for the preparation process of nanoformulation and nanoformulation-promoted intracellular delivery of protein.

In this work, as seen in Figure 1, the Fmoc ligand adjacent to L-arginine is substituted for functional DBCO containing alkynyl with a similar structure to Fmoc. DBCO contains two benzene rings and an electron conjugation structure, which provide the intermolecular force with hydrophobic and π-π stacking interaction. Thus, the DBCO-modified arginine derivatives are conducive to the formation of self-assembled materials through intermolecular interaction. In addition, crosslinker C, azide-functionalized triethylamine, was introduced to enhance and stabilize the morphological structure of the self-assembled nanovector formed by DBCO-modified L-arginine derivative via click chemical reaction, which avoids complicated preparation steps and the application of non-aqueous solvents. Saporin (SA), derived from saponaria officinalis seeds, was selected as the model toxic protein, which lead to the apoptosis of different cell lines by disrupting the bioactivity of cytosolic ribosome related to protein synthesis. The DBCO-modified arginine derivative was employed to load saporin via hydrophobic and electrostatic interaction, which was subsequently added with crosslinker C to form the stable DRC/SA nanoparticles. Furthermore, DRC/SA nanoparticles were coated with hyaluronic acid to shield their cationic toxicity and enhance intracellular saporin delivery efficiency via the recognition of CD44 overexpression on the cancer cell membrane, which induced the death of various cancer cell lines. Compared with DRC/SA, the DRC/SA/HA showed higher growth inhibition efficiency and lower IC_50_ value toward various cancer cell lines. Moreover, due to the properties of HA, such as its negative charges, biocompatibility and specific targeting, we hypothesize that the uniform DRC/SA/HA nanoparticles with an appropriate diameter have the potential to demonstrate excellent stability, good blood circulation and rapid accumulation for further cancer therapy without damaging normal cells in vivo.

## 2. Materials and Methods

### 2.1. Materials

6-amino-1-decanol was purchased from Yuanye Bio-technology (Shanghai, China). L-Arginine was purchased from Tansoole (Shanghai, China). Saporin (SA), Bovine serum albumin (BSA) and Hyaluronic acid (HA) were purchased from Sigma-Aldrich (St. Louis, MO, USA). Other chemical reagents were bought from Bidepharm (Shanghai, China). DAPI was obtained from Solarbio (Beijing, China). FITC-NHS ester was purchased from RuixiBio (Xi’an, China). Human cervical carcinoma cell line (HeLa), human breast cancer cell line (MDA-MB-231) and mouse breast cancer cell line (4T1) were purchased from BNCC (Zhengzhou, China).

### 2.2. Synthesis

The synthesis of Compound **1**: A mixture of Et_3_N (440 μL, 3.2 mmol) and 6-aminodecan-1-ol (374.4 mg, 3.2 mmol) was added to the solution of 4-nitrophenyl chloroformate-substituted DBCO (800 mg, 2.08 mmol) in CH_2_Cl_2_. The reactive solution was stirred at room temperature overnight, then concentrated under reduced pressure. The residue was dissolved in EtOAc (50 mL), and subsequently washed with saturated aqueous sodium bicarbonate (3 × 50 mL). The organic phase was added to Na_2_SO_4_ to reduce water, and condensed in vacuo. Then, the residue was purified via column chromatography with petroleum ether/ethyl acetate (*v*/*v* = 1:1) to achieve Compound **1**. ^1^H-NMR (400 NMR, CDCl_3_): δ 1.27–1.65 (m, 8H), 2.83–2.95 (dd, 1H), 3.05–3.34 (m, 3H), 3.54–3.71 (t, 2H), 5.40–5.57 (s, 1H), 7.27–7.51 (m, 8H). ^13^C-NMR (100 MHz, CDCl_3_): δ 25.33, 26.42, 29.98, 32.55, 40.96, 46.19, 62.75, 76.69, 109.98, 112.90, 121.29, 123.64, 125.94, 126.24, 127.03, 127.86, 128.02, 129.87, 151.00, 153.76, 155.41. FT-MS (ESI) m/z: [M + H]^+^ calculated 364.18, found 364.44.

The synthesis of Compound **2**: The combination of Et_3_N (132 μL, 0.96 mmol) and DMAP (117 mg, 0.96 mmol) was added to the solution of Compound **1** (300 mg, 0.81 mmol) in CH_2_Cl_2_. 4-nitrophenyl carbonochloridate (192 mg, 0.96 mmol) dissolved in CH_2_Cl_2_ was introduced dropwise into the mixture. After stirring at room temperature for 24 h, the reaction solution was condensed under reduced pressure and the residue purified via column chromatography with petroleum ether/ethyl acetate (*v*/*v* = 5:1) to obtain Compound **2**. ^1^H-NMR (400 NMR, CDCl_3_): δ 1.33–1.88 (m, 8H), 2.80–2.98 (dd, 1H), 3.03–3.34 (m, 3H), 4.16–4.37 (m, 2H), 5.43–5.59 (s, 1H), 7.27–7.56 (m, 10H), 8.19–8.36 (d, 2H). ^13^C-NMR (100 MHz, CDCl_3_): δ 25.32, 26.26, 28.39, 29.26, 29.90, 46.18, 69.38, 76.68, 109.91, 112.91, 121.31, 121.78, 123.59, 125.29, 125.97, 126.26, 127.05, 127.85, 128.02, 129.85, 145.32, 150.97, 151.61, 152.52, 155.42, 155.52. FT-MS (ESI) m/z: [M + K]^+^ calcd 567.19, found 567.63.

General procedure for the synthesis of compound DR: To the solution of Compound **2** (300 mg, 0.57 mmol) and Et_3_N (450 μL, 2.28 mmol) in THF (30 mL), L-arginine (198 mg, 1.14 mmol) dissolved in pH 8–9 H_2_O/THF was added. The reaction solution was stirred at room temperature overnight. After THF was removed in vacuo, the residue was added to EtOAc, then acidized with HCl (pH = 3). The organic phase was dried with Na_2_SO_4_ and concentrated under reduced pressure. The residue was purified via column chromatography, with CH_3_OH/EtOAc (*v*/*v* = 1:2) as the eluent used to achieve compound DR. ^1^H-NMR (400 MHz, CD_3_OD): δ 1.28–1.97 (m, 12H), 2.74–2.89 (dd, 1H), 3.02–3.23 (m, 5H), 3.97–4.18 (m, 3H), 5.35–5.46 (s, 1H), 7.21–7.65 (m, 8H). ^13^C-NMR (100 MHz, CD_3_OD): δ 24.99, 25.16, 26.00, 28.53, 28.63, 29.34, 40.29, 40.50, 45.74, 53.40, 64.70, 76.38, 109.59, 112.41, 120.97, 123.52, 125.49, 125.76, 126.86, 127.84, 127.93, 129.59, 151.02, 152.28, 156.62, 157.11, 157.63. FT-MS (ESI) m/z: [M + H]^+^ calcd 564.27, found 564.69.

### 2.3. Preparation and Characterization of DRC/Protein Nanoformulations

The synthesized DR monomer was first dissolved in sterilized ultrapure water with a concentration of 1 mg/mL, then mixed with protein solution (dissolved in sterile water, 1 mg/mL) at a weight ratio of 4:1 for BSA (DR:BSA) and 5:1 for saporin (DR:SA) for 10 min at room temperature, respectively. After the addition of crosslinker C (DR/C, molar/molar, 3:1), DRC/protein nanoformulations were obtained through the click chemical reaction. After that, DRC/protein gently mixed with HA (DR/HA, *w*/*w*, 1/5) to form the complexes DRC/BSA/HA and DRC/SA/HA. The size and zeta potential of each sample of DRC/protein nanoformulations were measured using dynamic light scattering (DLS) at room temperature (Malvern Zetasizer Nano ZS 90, Marvin, UK). All DRC/protein samples were deposited separately onto a 200-mesh carbon-coated copper grid and air-dried before observation. The morphologies of DRC, DRC/BSA, DRC/BSA/HA and DRC/SA/HA were observed via transmission electron microscopy (TEM, HT7700, HITACHI, Tokyo, Japan). Agarose electrophoresis was used to assay the formation of DRC/protein complexes. The various complexes (fixed BSA or SA at a dosage of 5 μg) were electrophoresed on the 1% (*w*/*v*) agarose electrophoresis for 1 h at the DR/protein/HA weight ratio of 4:1:20 for BSA or 5:1:25 for SA; an image was then taken by the gel imaging instrument (ImageQuant™ LAS 500) after Coomassie blue staining.

### 2.4. Protein Loading Efficiency

A total of 10 μg BSA or SA (1 mg/mL) was added into DR aqueous solution (1 mg/mL) at the weight ratio of 1:3 (protein/DR, *w*/*w*, 1:3), respectively. Then, the crosslinker C was added into the mixture solutions of DR/BSA and DR/SA with a corresponding molar ratio (DR/C, molar/molar, 3:1) and stirred for 10 min. Subsequently, the supernatant was obtained through centrifugation at 8000 rpm for 5 min, then tested for the unloaded BSA or SA via a BCA Protein Assay Kit according to the manufacturer’s instructions. The protein loading efficiency by DRC was calculated through the following equation [39]: Loading Efficiency = ((total weight amount of protein—the weight amount of unloaded protein)/ the total weight of nanoformulations) × 100.

### 2.5. The Stability of Nanoformulations

A total of 500 μL DR (1 mg/mL) aqueous solution was added to BSA or SA (1 mg/mL) at a weight ratio of 4:1 for DR/BSA and 5:1 for DR/SA. After the introduction of crosslinker C, the DRC/BSA and DRC/SA were prepared at a DR/C molar ratio of 3:1. Then, after the addition of HA with a weight ratio of 4:1:20 for DR/BSA/HA and 5:1:25 for DR/SA/HA, the samples’ DRC, DRC/BSA/HA and DRC/SA/HA diameter were prepared in order to measure their stability in the aqueous solution every 6 h for 30 h at room temperature using DLS. The circular dichroism spectra (CD) were applied to estimate the conformation of free SA and DRC/SA/HA. CD spectra were measured through using the Bio-Logic MOS-450 Spectropolarimeter at r.t. within the wavelength range of 190–250 nm under N_2_ atmosphere.

### 2.6. Synthesis of FITC-Labeled BSA (BSA-FITC) and FITC-Labeled Saporin (SA-FITC)

For the synthesis of BSA-FITC and SA-FITC, 2 mg BSA or SA were dissolved in phosphate-buffered saline (PBS, pH 8.0) to obtain the protein concentration of 1 mg/mL, and the solution was added to FITC-NHS ester dissolved in a DMSO at protein/FITC-NHS ester molar ratio of 1:7 and solution volume ratio of 1:1 overnight at 4 °C conditions. The product was purified via intensive dialyzation with distilled water (3500 Da) and then lyophilized to obtain BSA-FITC and SA-FITC, which were added to sterile water to prepare the protein–FITC solution at a final concentration of 1 mg/mL. The BSA and SA solution were stored at 4 °C before further use.

### 2.7. Cell Culture and Cellular Uptake

MDA-MB-231 were cultured in Leibovitz’s L-15 Culture Medium, comprising 10% (*v*/*v*) fetal bovine serum (FBS, GIBCO) and 1% 100 U/mL penicillin sulfate and streptomycin (P/S) in an incubator at 37 °C. HeLa and 4T1 were cultured in Dulbecco’s modified Eagle’s medium (DMEM, GIBCO) containing 100 U/mL P/S and 10% (*v*/*v*) FBS at 37 °C in a humidified atmosphere of 5% CO_2_. The cellular uptake efficiency of FITC-BSA/FITC-SA loaded nanoformulations was measured. Briefly, HeLa and MDA-MB-231 cells were seeded in 96-well plates at a density of 1 × 10^4^ cells per well and incubated at 37 °C for overnight until 80% confluence. The cells were washed twice with PBS, and then 100 µL fresh DMEM containing BSA-FITC, DRC/BSA-FITC or DRC/BSA-FITC/HA was added separately into Hela cells to maintain the BSA-FITC concentration of 10 μg/mL at DR: BSA-FITC: HA weight ratio of 4:1:25 for another 8 h of incubation [40]. In addition, the fresh medium including DRC/SA-FITC, DRC/SA-FITC/HA (DR: SA-FITC: HA, *w*:*w*:*w*, 5:1:25) was added into MDA-MB-231 cells with an SA concentration of 10 μg/mL for the same duration. The cell nuclei were stained with DAPI (5 mg/mL) and the images were measured with a confocal laser scanning microscope. Then, the cells were harvested and dispersed in PBS for quantitative analysis via flow cytometry (BD FACS Calibur).

### 2.8. Cytotoxicity Assay

The cytotoxicities of DRC and DRC/HA complexes were measured via CCK-8 reduction assay. Generally, 4T1, MDA-MB-231 and HeLa cells were seeded at a density of around 1 × 10^4^ cells per well in 96-well plates overnight. The cells were washed twice with PBS and cultured with 100 μL of the medium containing DRC and DRC/HA at different concentrations (0–160 µg/mL) for 8 h. After that, the incubation medium was discarded and the cells were further incubated with fresh medium containing 10% FBS and 1% P/S for 20 h. Then, the cell viability was measured using a cell counting kit (CCK-8) assay according to the introductions supplied by the manufacturer. Five repeats were conducted for each sample.

### 2.9. In Vitro Anticancer Activity

4T1, MDA-MB-231 and HeLa cells were seeded in 96-well plates at a density of 1 × 10^4^ cells per well a day before experiment, and then cells were treated with different concentrations of free SA, DRC/SA and DRC/SA/HA complexes (the concentration of saporin varied from 0 to 80 × 10^−9^ M) for 8 h of incubation. Then, the cultured medium was replaced by the fresh complete medium containing 10% FBS and 1% P/S for another 48 h incubation. The cell viability was also measured by the CCK-8 assay, and the percentage of cell viability was calculated by comparing the absorbance of the control cells to that of free SA, DRC/SA and DRC/SA/HA complex-treated cells, respectively [41].

### 2.10. Statistical Analysis

All data were expressed as the mean ± standard deviation (SD). All samples were examined with repeats. The Graphpad Prism 9.0 and Origin 2019b software was utilized for statistical analysis.

## 3. Results and Discussion

### 3.1. Synthesis the of Vector and Crosslinker

**Figure 2:**
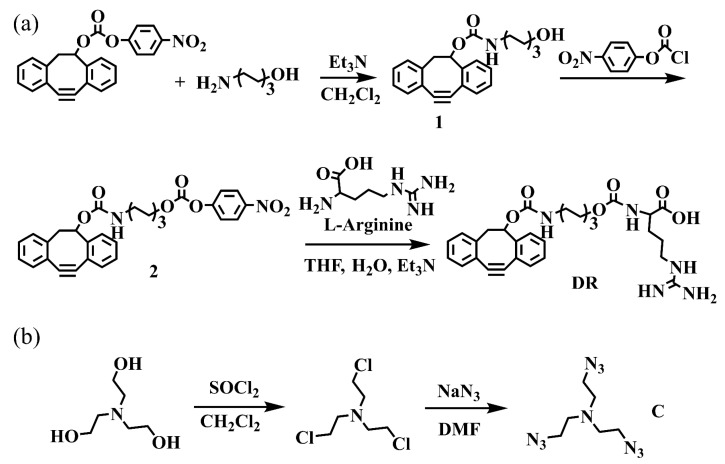
(**a**) The synthesis process of the L-arginine derivative functionalized by DBCO. (**b**) The synthesis route of crosslinker C.

In this study, (Figure 2a) DBCO-modified p-nitrophenylcarbonate was reacted with aminohexanol to obtain compound **1**. Subsequently, Compound **1** was modified with p-nitrophenyl chloroformate to obtain Compound **2**, which was further added with L-arginine to obtain the final DR molecules. The hydrophobic nature of DBCO in DR was combined with the hydrophilic nature of L-arginine to endow the DR with amphiphilic properties, which was conducive to the self-assembly of DR in the aqueous solution. In addition, as seen in Figure 2b, the hydroxy of triethanolamine was replaced with chlorine, then reacted with azide to obtain the multi-claw azide functionalized triethylamine (crosslinker C). The crosslinker C was integrated into nanoformulation formed by amphiphilic DR to enhance its self-assembly and stabilize the morphology of DR via the click chemical reaction. Compounds related to the main synthesis route were characterized and confirmed by NMR (nuclear magnetic resonance) and MS (mass spectrometry).

### 3.2. Preparation and Characterization of Various Nanoformulations

**Figure 3:**
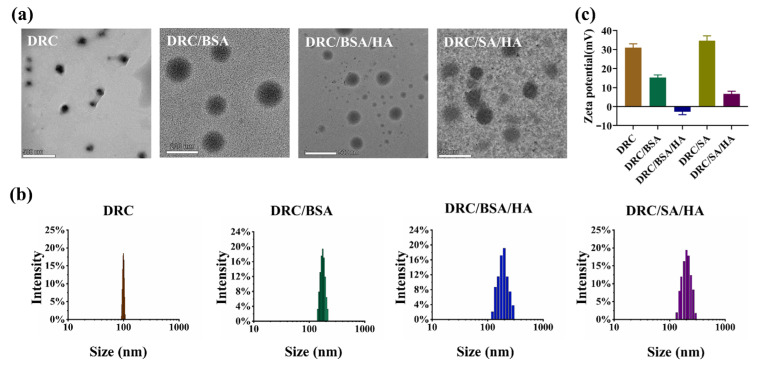
Characterization of various nanoformulations with a final DR concentration of 1 mg/mL. (**a**) TEM images of DRC, DRC/BSA, DRC/BSA/HA and DRC/SA/HA. (**b**,**c**) The hydrated diameter distribution and Zeta potential of various nanoformulations measured using DLS.

The combination of the DBCO-modified arginine and crosslinker C with azide was employed as a building block for the formation of self-assembled nanoparticles enhanced by the click chemical reaction. The detailed process was performed as follows: To evaluate the protein loading ability of DR based on electrostatic interaction, bovine serum albumin (BSA, pI = 4.7) was chosen as a model protein. The amphiphilic DBCO-modified L-arginine derivative dissolved in aqueous solution was loaded with model protein BSA and subsequently added to crosslinker C to improve loading efficiency and stabilize the self-assembly of BSA-loaded nanoparticles (DRC/BSA). After calculation, the loading efficiency of DRC to BSA was 20%. As is shown in Figure 3a,c, the results obtained by dynamic light scattering (DLS) indicated that the mean hydrated particle diameter of nanopreparation DRC was 100 nm, and the zeta potential of DRC was 30 mV. Furthermore, the results from transmission electron microscopy (TEM) demonstrated that DRC had a spherical morphology, and the diameter size of DRC nanoparticles stayed the same with the data of DLS (Figure 3b). The nanoparticle size of BSA-loaded DRC increased to 175 nm compared with that of DRC. Meanwhile, the average zeta potential of DRC/BSA decreased to 15 mV, indicating that the BSA was successfully embedded into the structure of DRC by electrostatic interaction. After the introduction of HA into the DRC/BSA solution, the DLS data show that DRC/BSA/HA with a 200 nm size was bigger than DRC/BSA, but the outer potential of DRC/BSA/HA decreased to −3 mv, illustrating that HA-coated DRC/BSA was promoted by electrostatic interaction.

**Figure 4:**
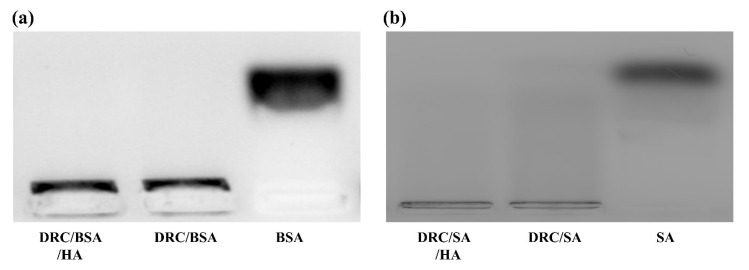
Protein loaded in DRC-based nanocomposites detected through agarose gel electrophoresis. (**a**) The loading situation of BSA in DRC/BSA and DRC/BSA/HA nanoformulations. (**b**) The loading situation of SA in DRC/SA and DRC/SA/HA nanoformulations.

For saporin (SA, pI = 9.5) [42], the DRC/SA was successfully obtained according to the DRC/BSA preparation process. The loading efficiency of DRC to SA was 16.7%. The nanoparticles encapsuling SA were coated with hyaluronic acid to form the final nanopreparation DRC/SA/HA. The results in Figure 3a,b indicate that the diameter size of the DRC/SA coated with HA increased to 230 nm compared to DRC, with an average size of 100 nm. However, the mean potential of DRC/SA slightly increased to 32 mV compared with the zeta potential of 30 mV on the surface of DRC (Figure 3c), which demonstrated that the SA was integrated into the morphology structure of DRC via hydrophobic interaction. The average potential of DRC/SA/HA decreased to 5 mV because of the electrostatic interaction between HA and DRC/SA. As is shown in Figure 4a,b, the protein loading ability of DRC-based nanoparticles was also evaluated through agrose gel electrophoresis, and BSA was completely loaded into DRC/BSA and DRC/BSA/HA at a DR/BSA/HA weight ratio of 4:1:25. For saporin protein, SA was completely loaded in DRC/SA and DRC/SA/HA at the weight ratio of 5:1:25 (DR/SA/HA, 5:1:25). The above results indicate that DRC-based nanomaterials were able to load with protein to form nanoformulations with suitable diameter for the intracellular delivery of cargo via electrostatic and hydrophobic interaction.

### 3.3. Stability of the Protein-Loaded Nanoparticles

It was crucial for efficient cytosolic protein delivery to maintain the stability of the DRC-based nanopreparation loaded with protein in the aqueous solution. Moreover, during the process of protein delivery, protein was successfully transported into cells without changes in bioactivity, which is important for protein-based biotherapeutics in diseases. Therefore, it was necessary to evaluate the stability of nanoparticles and bioactivity of protein.

**Figure 5:**
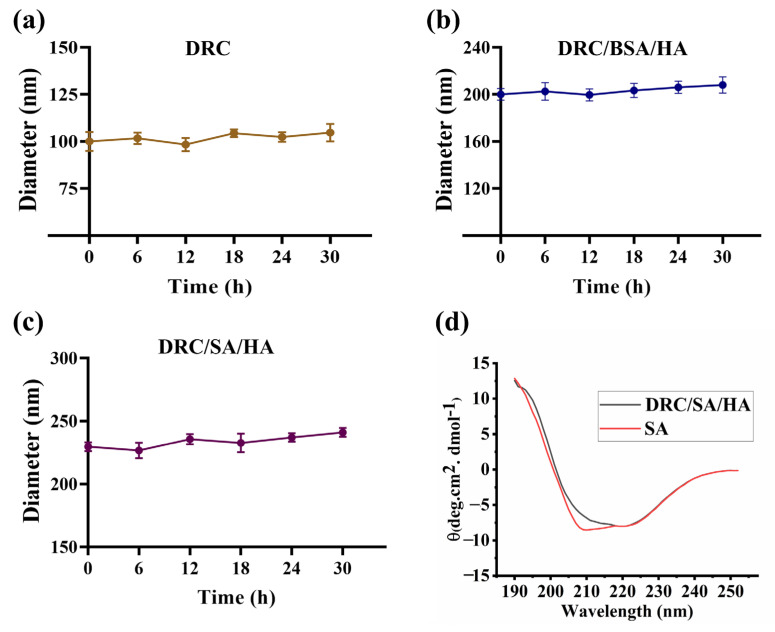
Stability of DRC, DRC/BSA/HA and DRC/SA/HA in deionized water. (**a**–**c**) The diameter value was measured within 30 h at room temperature. (**d**) CD spectra of free SA and DRC/SA/HA complexes.

The stability of DRC-based nanoparticles was investigated at room temperature. Figure 5a–c shows that particle size changes slightly in water, demonstrating the good stability of DRC-based nanoparticles. Furthermore, as shown in Figure 5d, compared to free SA, the delivery carrier and HA have little effect on the conformation of protein, maintaining the bioactivity of SA. The above results indicated that DRC-based nanoparticles loaded with protein have many advantages in cytosolic protein delivery in vitro or in vivo.

### 3.4. Cellular Uptake of Proteins

**Figure 6:**
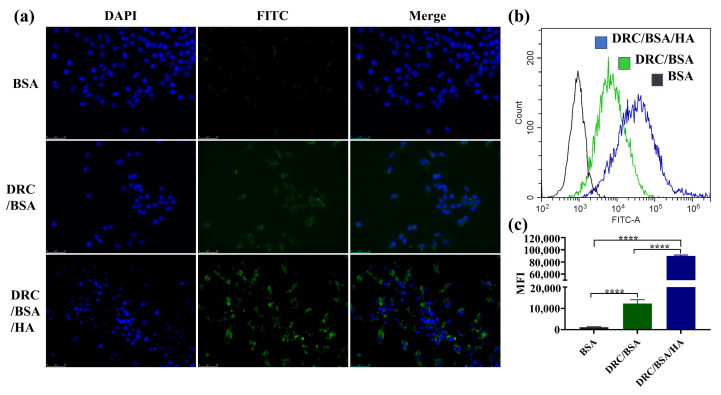
In vitro Hela uptake of BSA. (**a**) Fluorescence confocal image of Hela cells after 8 h of incubation with free BSA-FITC, DRC/BSA-FITC and DRC/BSA-FITC/HA, respectively. Scale bar, 75 μm. (**b**,**c**) Flow cytometry plots (**b**) and statistical data (**c**) of Hela for evaluating the intracellular delivery efficiency of BSA. Data are presented as mean ± SD. **** *p* < 0.0001.

**Figure 7:**
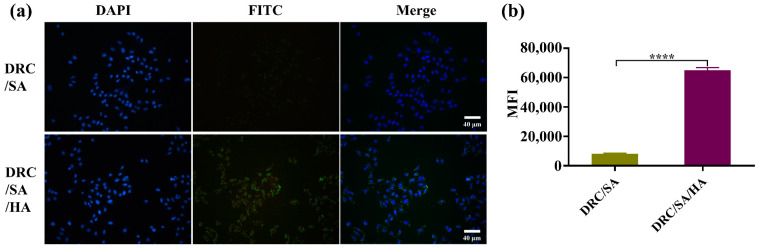
DR-based supramolecular nanoformulations for cytosolic saporin delivery. (**a**) CLSM images of MDA-MB-231 cells treated with DRC/SA and DRC/SA/HA for 8 h. Scale bar, 40 μm. (**b**) Mean fluorescence intensity of the treated MDA-MB-231 cells using flow cytometry. The data are presented as mean ± SD. **** *p* < 0.0001.

For protein drugs acting on targets inside cells, it is crucial to achieve intracellular protein delivery without decreased bioactivity of the protein [43]. Therefore, the internalization efficiency of DRC-based nanoformulation into cells should be precisely evaluated. To observe the cytosolic distribution of DRC-based self-assembled nanoparticles loaded with protein, BSA and saporin were combined with FITC to track their location in real time. Nanoparticles loaded with BSA-FITC or saporin-FITC were incubated with the medium of Hela or MDA-MB-231, respectively. Subsequently, the cell lines were observed and analyzed through fluorescence confocal microscopy and flow cytometry. As is shown in Figure 6a, less fluorescence of BSA-FITC was observed in the cytosol of Hela cells, which indicated that free BSA was difficult to internalize into Hela cells. However, the results of DRC/BSA group shown that moderate BSA could penetrate into Hela cells with the DRC-assisted endocytosis pathway. After the addition of HA to the DRC/BSA, the strong fluorescence of the DRC/BSA/HA group was detected via CLSM, indicating that DRC/BSA/HA was efficiently internalized into the cytoplasm of Hela cells with CD44-receptor-mediated endocytosis. According to the flow cytometry data in Figure 6b,c, the results demonstrated that the mean fluorescence intensity of DRC/BSA/HA was more than eight-fold that of DRC/BSA, which was consistent with the consequence of fluorescence confocal microscopy. In addition, as shown in Figure 7a, the fluorescence of saporin-loaded DRC was weakly detected by CLSM, manifesting that limited saporin could be taken up by MDA-MB-231 cells via DRC-assisted endocytosis. Compared with DRC/SA, the HA-coated DRC/SA could efficiently enhance the cytosolic delivery efficiency of saporin by the CD44 receptor bound with HA (Figure 7b). The flow cytometry analysis data revealed that the mean fluorescence intensity of DRC/SA/HA was more than six-fold that of DRC/SA, which remained consistent with the results of CLSM. After saporin was ingested by cells, the uptake efficiency of protein by cells influenced the bioactivity of cytosolic ribosome related to the normal operation of cellular life. DRC-based direct delivery of saporin into the cytosol could increase apoptosis efficiency of cancer cells, which proves that DRC is a promising potential tool for intracellular protein delivery.

### 3.5. Cytotoxicity Evaluation of DRC and Anticancer Ability of DRC-Based Nanoformulations Containing Saporin

**Figure 8:**
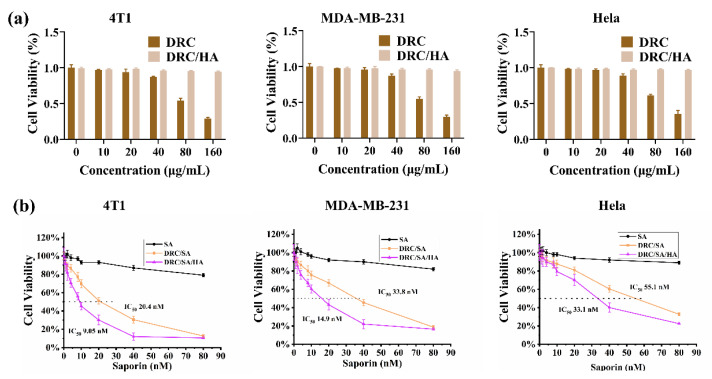
Cytotoxicity and anticancer ability of nanoformulations on different cell lines. (**a**) The viability of 4T1, MDA-MB-231 and Hela cells treated with DRC or DRC/HA at different concentrations. (**b**) The cytotoxicity of SA, DRC/SA and DRC/SA/HA with different concentrations on 4T1, MDA-MB-231 and Hela cells. Data are calculated as the mean ± SD (*n* = 5). In the cell viability assay, the weight ratio of DR/SA/HA was 5:1:25.

In order to understand the biocompatibility of DRC and HA-coated DRC (DRC/HA), cytotoxicity was investigated in three different cell lines from humans and mice using a CCK-8 assay kit. In human cells, different types of cancer cells, namely MDA-MB-231 (a breast cancer cell line) and Hela (a human cervical carcinoma cell line), were selected to study the cytotoxicity of the nanoformulations. As shown in Figure 8a, the cell survival results showed that DRC had significant cytotoxicity at concentrations above 80 μg/mL. In contrast, after the introduction of HA, DRC/HA had no significant toxicity toward three different cell lines even if the concentration reached up to 160 μg/mL. The main reason was that partly positive charges of DRC were blocked with the introduction of HA, which greatly reduced the cytotoxicity of DRC. Generally, the above data proved that DRC/HA had good biocompatibility and could be used as a cytosolic delivery tool for protein.

Based on the above results, we then evaluated the in vitro anticancer efficacy of protein-loaded DRC/SA/HA nanoparticles on different cell lines. Saporin, famous for the ability to block protein synthesis in eukaryotic cells by disrupting the specific nucleotide in ribosomes, was selected as the therapeutic protein for cancer therapy [44]. As shown in Figure 8b, free saporin did not cause any significant cytotoxicity because of its poor cell membrane permeability. In contrast, DRC/SA displayed effective cell growth inhibition, and the half maximal inhibitory concentration (IC_50_) was 20.4 nM for 4T1 cells, 33.8 nM for MDA-MB-231 cells and 55.1 nM for Hela cells, respectively. Meanwhile, CD44-targeted DRC/SA/HA exhibited higher growth inhibition efficiency than nontargeted DRC/SA toward the different cancer cells with lower IC_50_ (9.05 nM for 4T1 cells, 14.9 nM for MDA-MB-231 cells and 33.1 nM for Hela cells) via recognition of the over-expressed CD44 receptor on the cell membrane. Altogether, these data convincingly prove that DRC/SA/HA can efficiently enhance the anticancer effect.

## 4. Conclusions

In summary, we prepared the DBCO-modified L-arginine derivatives (DR) by replacing Fmoc neighboring L-arginine with DBCO groups similar to Fmoc. DR were regarded as basic building blocks, which were combined with azide-functionalized triethylamine (crosslinker C) via click chemical reaction to construct the DRC-based self-assembled supramolecular nanovector for delivering cargo proteins, such as BSA and saporin, into the cytosol of various cell lines. The results indicated the L-arginine derivatives functionalized by DBCO were efficiently loaded with BSA or saporin to form nanoparticles with uniform and moderate diameters, which was conducive to the nanoparticle-assisted cellular endocytosis of proteins. The DRC/SA was coated with hyaluronic acid to obtain good biocompatibility and promote intracellular protein delivery efficiency, which led to the excellent apoptosis of various cancer lines. This study provides a novel approach for developing and designing DRC-based supramolecular nanomaterials for cytosolic protein delivery in potential cancer therapy.

In the future, DRC-based vectors could be utilized to load mRNA via the electrostatic interaction between guanidyl and phosphate groups to develop nanovaccines to stimulate the immune response in order to protect against the occurrence of various diseases, such as cancer, bacterial infection, virus infection and Alzheimer’s disease. In addition, we hypothesize that, through metabolism engineering with N-azidoacetylgalactosamine-tetraacylated (Ac4GalNAz), the chemical receptor azide is introduced onto the cell membrane or tumor tissue. Subsequently, DBCO-modified amino acids loading drugs can target and penetrate into the engineered cells with chemical receptor azides via click chemical reactions. Consequently, we believe that DBCO-functionalized amino acid derivatives show a great promise for drug delivery.

## Data Availability

Not applicable.
